# The importance of evolutionarily conserved C‐terminal basic residues for the stability of proapoptotic Bax protein

**DOI:** 10.1002/2211-5463.12096

**Published:** 2016-08-25

**Authors:** Jorge L. Rosas‐Trigueros

**Affiliations:** ^1^ Laboratorio Transdisciplinario de Investigación en Sistemas EvolutivosSEPI de la ESCOM del Instituto Politécnico NacionalMéxico DFMéxico

**Keywords:** apoptosis, molecular dynamics, protein stability

## Abstract

Bax is a protein that promotes apoptosis (a form of cell death). The atomistic details of the mechanism by which Bax is activated during apoptosis remain a subject of debate. C‐terminal basic residues in the sequence of Bax show remarkable conservation across a variety of species. The role of these charged residues in the stability of Bax was investigated by submitting substituted mutants to molecular dynamics simulations at high temperatures. Mutation of either or both K189 and K190 led to dramatic changes in helical content, radius of gyration, proximity of the C terminus to the core of the protein, exposure of the BH3 domain, and bundling of the core. These results suggest a critical role of positively charged residues close to the C terminus in the structural stability of Bax.

**Abbreviations**
AA: K189A/K190A double point mutant
aH: alpha‐helix
AK: K189A single point mutant
BH: Bcl‐2 Homology domain
DCT: C‐terminal deletion mutant of Bax
DPM: double point mutant
EE: K189E/K190E double point mutant
KA: K190A single point mutant
Lp: Loop
LpNt: N‐terminal loop
MD: molecular dynamics
MM: K189M/K190M double point mutant
r189r190: residues 189 and 190
rDD: residues D68 to D98
Rg: radius of gyration
RMSD: root mean square deviation
RMSF: root mean square fluctuation
SASA: solvent accessible surface area
SPM: single point mutant
WT: wild‐type monomeric human Bax

Proteins of the Bcl‐2 family play an essential role in the mechanism of the cell death program called apoptosis 1. The known proteins in this family have either one or four Bcl‐2 Homology (BH) domains and can be grouped into three classes: (a) the proapoptotic multidomain proteins with four BH domains (BH1‐4); (b) the antiapoptotic members, also with BH1‐4; and (c) the proapoptotic BH3 only proteins. The BH3 domain, common to all the proteins in the Bcl‐2 family, has been found to participate in the homo‐ and heterodimerization of Bcl‐2 family proteins 2. Bax (192‐aa) is a proapoptotic member of the Bcl‐2 family (BH1‐4) 3. In its native conformation, Bax is a globular, soluble protein and is found in the cytosol 4, 5. In response to apoptotic signals, Bax undergoes a conformational change which causes the migration of this protein to mitochondria from cytosol. Bax oligomers form pores on the outer membrane, resulting in the release of at least five apoptogenic proteins such as cytochrome c from the mitochondrial intermembrane space which contribute to the degradation of cell structures in different ways 6. The solution structure of Bax shows nine alpha helices (aH1‐9), joined by loops (Lp1‐2, Lp2‐3, Lp3‐4, Lp4‐5, Lp5‐6, Lp6‐7, Lp7‐8 and Lp8‐9) with some turns and a large N‐terminal loop (LpNt) (Fig. 1) 5. The alpha‐helices are bundled in a fold common to several Bcl‐2 family proteins known as the Bcl‐2 core 2.

**Figure 1:**
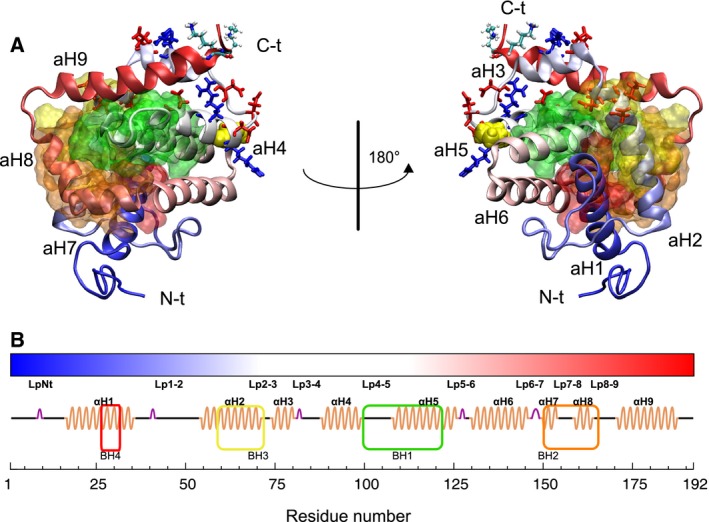
Bax 3D structure (PDB ID: 1F16). (A) Cartoon representation of Bax. The BH domains are represented with surfaces colored green (BH1), orange (BH2), yellow (BH3), and red (BH4). Residues 189 and 190 are shown in Licorice and CPK representations, respectively, both colored by atom name. P88 (the central residue in rDD) is shown in VDW (yellow). Charged residues in rDD within 0.7 nm of residues 189 and 190 are shown in Licorice colored in red (negative) and blue (positive). (B) Color coding and labels of the protein regions (alpha‐helices and loops) used throughout this work. The color of the peptidic chain transitions from blue in the N terminus to red in the C terminus. Labels indicate the N‐terminal (N‐t) and C‐terminal (C‐t) ends of the protein and the alpha helices (aH1‐9).

Computational modeling and simulation studies of the Bcl‐2 family have provided knowledge of the structural details involved in the stability and function of these proteins, as previously reviewed 7. For instance, the highly hydrophobic aH9 is accommodated in a hydrophobic groove and covers residues that are likely to be relevant for the function of the protein, due to their high level of conservation among similar sequences 8. This result suggests that the active conformation might include a displacement of aH9. A previous molecular dynamics (MD) study of Bax at high temperatures found that aH9 shows a tendency to separate from the core of the protein. Such conformational change seems to be prevented by electrostatic interactions involving lysines close to the C terminus (K189, K190), suggesting that the perturbation of those interactions could be among the early steps in the mechanism of Bax activation, as reported previously 9. The presence of positively charged residues (lysine or arginine) close to the end of the protein is common to nearly all Bcl‐2 family members 10. The conservation of these residues is remarkable for Bax across a variety of species (Fig. S1, Table S1).

Studies focusing on the C‐terminal domain of Bax have investigated its role on membrane affinity, interaction with other proteins, and proapoptotic function 10, 11, 12, 13, 14. Besides, the removal of this domain has been shown to destabilize the protein 15. MD simulations of full‐length Bax have been reported, but point mutations near the C terminus have not been considered 9, 15, 16, 17, 18. Particularly, the role of residues K189 and K190 on the structural stability of Bax has not been addressed. These lysines are close to Lp3‐4 in the native conformation. Lp3‐4 is in the middle of a region with abundant charged residues. This region goes from D68 to D98 (rDD) and includes three residues with positive (R78, R89 and R94) and eight with negative charges (D68, E69, D71, E75, D84, D86, E90 and D98). Some of the latter have been shown to interact with K189/K190 in MD simulations at high temperatures as reported previously 9.

Despite considerable attention focused on this protein, the atomic‐level description of the activation of Bax remains elusive. To test the hypothesis that mutations in lysines 189 and 190 alter the stability of Bax, MD simulations at different temperatures were performed on wild‐type monomeric human Bax (WT) and five mutants. The mutations considered were the K189A (AK) and K190A (KA) single point mutants (SPMs) and the K189A/K190A (AA), K189E/K190E (EE), and K189M/K190M (MM) double point mutants (DPMs). The side‐chain of lysine is large and positively charged. In contrast, alanine possesses a small, nonpolar side‐chain. The KA, AK and AA variants were designed to abolish the hypothetical stabilizing function of K189 and K190. The MM and EE DPMs were studied *in vitro* by Arokium *et al*. 10. It was thus interesting to study these two variants as well. The results suggest that all mutations considered decrease the protein stability. The mutations affect helical content, radius of gyration, proximity of the C terminus to the core of the protein and BH1‐4 bundling. The analyses of the observed behavior might contribute to advance our understanding of the conformational changes involved in the activation of Bax.

## Materials and methods

All MD simulations were performed with Gromacs 4 19, using the OPLS‐AA force field 20. The starting coordinates for MD simulations of the wild‐type Bax monomer (WT) were obtained from the first NMR model located in the Protein Data Bank (PDB ID: 1F16) 5. The mutants were constructed from the starting coordinates using the software pymol (http://pymol.sourceforge.net/). Hydrogen atoms were added to the system by means of the Gromacs pdb2gmx routine. The leap‐frog algorithm for integrating Newton equations was used, and periodic boundary conditions were applied. The proteins were solvated in rhombic dodecahedral boxes of SPC water 21, with a minimum distance of 1 nm from the protein to the edge of the box (Table S2). To obtain a neutral total charge in the system, Na+ counterions were added. Further details can be found in Table S3. During energy minimization, the steepest descents algorithm was used and the convergence was reached in less than 200 steps. Further equilibration of the system was accomplished in 5000 steps (10 ps) of MD with restricted protein atoms and NVT conditions at 310 K. Afterward, 20 ns of production MD were obtained at 310 K. Using the latter trajectory as a starting point, another 20 ns of production MD were obtained at 400 K, where the system was gradually heated during the first 500 ps. Finally, the trajectory at 400 K was used as a starting point for further 20 ns, where the system was heated from 400 to 500 K during the first 500 ps. NPT conditions for production MD simulations, bond restriction with LINCS, periodic boundary conditions, and PME electrostatics were configured as reported previously 9. The interesting behavior of the AA mutant at 500 K described below motivated the extension of this simulation for another 20 ns. The shortest periodic distance is above 1.9 nm for all simulations. Only one simulation per system was performed.

## Results and Discussion

### Overall stability

**Figure 2:**
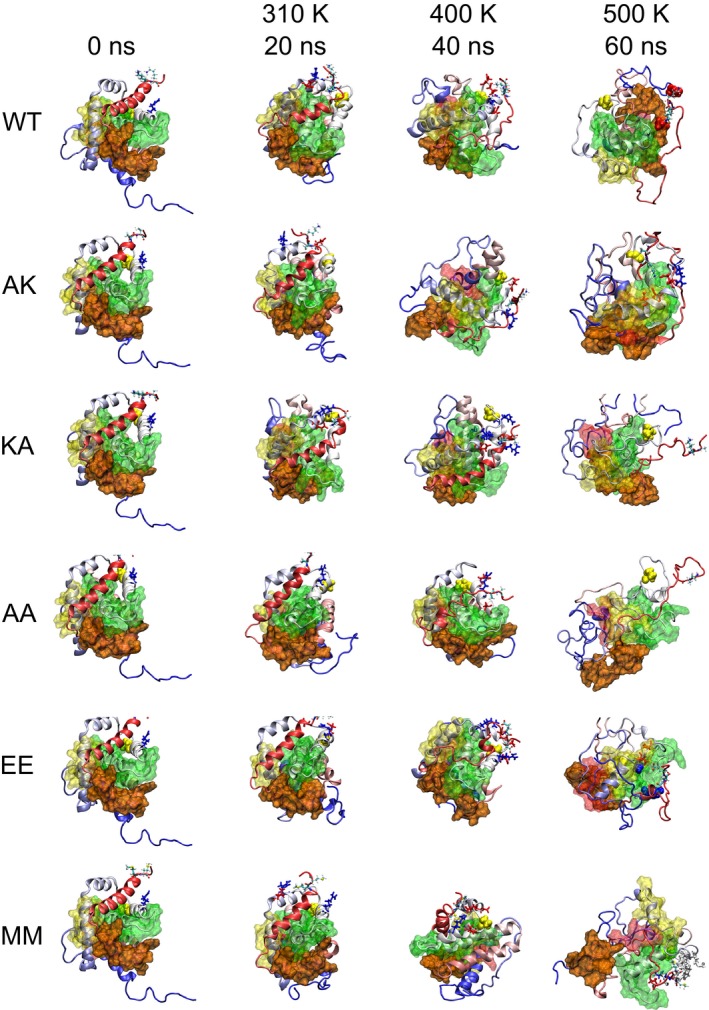
Snapshots of the protein structures at different time points at different temperatures. Color coding is the same as described in Fig. 1. Charged residues in rDD within 0.7 nm of residues 189 and 190 (Table S5) are shown in Licorice colored in red (negative) and blue (positive).

The alpha‐carbon root mean square deviation (RMSD) values calculated for the simulations show, in general, an initial increase and decreasing fluctuations as stable conformations are reached by the proteins (Fig. S2). LpNt is separated from the globule in the starting coordinates and moves toward the core producing relatively large values of RMSD for all proteins at 310 K. This behavior correlates with decreases in radius of gyration and solvent‐accessible surface area, as discussed below, and is in full agreement with previously reported results 9. The RMSD values are bounded below 0.7 nm for the simulations at 310 K, below 0.9 nm at 400 K and below 1.7 nm at 500 K. Abrupt increases and decreases often appear due to unfolding or refolding of helices. These conformational changes are described in the following section. Root mean square fluctuation (RMSF) values are consistent with previously reported data, with BH2 as the BH domain with the largest mobility (Fig. S3) 9. The final structures from the MD simulations are shown in Fig. 2. Analyses of the complete trajectories show that these structures can be used to illustrate the loss of helical content and the BH1‐4 unbundling (sections 3.2 and 3.6, respectively). The PDB files for these structures are provided as supplementary material.

### Helical content

Noteworthy changes were encountered in the secondary structure of all mutants even at 310 K, where alpha‐helices 4, 7 and 8 unfold. aH4 refolds in all mutants at the end of the MD simulation at 310 K, whereas aH7 and aH8 remain unfolded in some cases as described below. In contrast, only aH8 unfolds in the WT protein at 310 K, but it refolds soon after (Fig. S4). All proteins in our simulations show a similar percentage of helical structure (alpha‐, 3‐ or 5‐helix) at 310 K (above 50%) and 400 K (mean value between 40% and 50%). At 500 K, the mutant proteins show a larger loss of helicity than the WT (Fig. S5, Table S4). The most dramatic decrease is observed for the AA mutant, where the minimum values are below 5%. Table 1 shows the alpha‐helices that have at least one turn (four residues) conserved in the final structures of the simulations performed. At 310 K, the WT protein retains at least one turn in all of its alpha‐helices while mutants AK, KA, and MM show the loss of either aH7 or aH8. The C‐terminal end of Bax is also affected at 400 K, with loss of structure in either of aH7‐9 for all proteins and all three alpha‐helices for the AA mutant. The small aH3 is also lost in the AK and KA SPMs. As expected, the largest loss of structure is seen at 500 K, but with remarkable differences between the WT protein and the variants. While the WT retains some alpha‐helical content in five alpha‐helices (aH2‐6), all alpha‐helices are lost for the KA mutant, only one is kept for the AA and EE DPMs and three remain in the AK and MM mutants. It is of note that aH2 (BH3) is among the most resilient domains together with aH5 (BH1) as their alpha‐helices are conserved in most cases at 500 K, whereas BH2 (aH7‐8) and aH9 appear to be the least stable because their alpha‐helical structure is lost for all proteins at 500 K. Also of note, BH4 (aH1) shows structural resilience in the MM mutant. These results show that the WT structure is more stable than all mutants considered and reveal the important role of K189 and K190 in the structural stability of Bax.

**Table 1:** Conserved alpha‐helices in the final structures. The number of the helix is shown if at least one turn is conserved (four residues)

Protein	310 K	400 K	500 K
WT	123456789	12345678*	*23456***
AK	1234567*9	12*456**9	*2**56***
KA	123456*89	12*4567*9	*********
AA	123456789	123456***	***4*****
EE	123456789	12345678*	*2*******
MM	1234567*9	123456**9	12**5****

Arokium *et al*. 10 found that the EE mutant has a high binding capacity to mitochondria and high cytochrome c‐release activity *in vitro*. In the MD simulations, the EE mutant has similar but lower stability than the WT protein at 310 and 400 K (Table 1, Fig. S4). In the native structure, K189 and K190 are located in the proximity of rDD, a region where about one‐third of the residues possesses a net electric charge (three positive, eight negative). Four negatively charged residues in this region were previously reported to interact with K189/K190, thus keeping these lysines close to the core 9. Substitution of K189/K190 with negatively charged residues allows these residues to interact with positively charged residues in rDD. The minimum distance between E189/E190 and R78/R94 remains below 0.3 nm for 91% of the time at 310 K, and below 0.2 nm for 74% and 56% of the time at 400 and 500 K, respectively (Fig. S6). This behavior indicates favorable electrostatic interactions arise between the negatively charged C‐terminal residues and positively charged residues in rDD. These interactions keep aH9 close to the core in a similar manner as for the WT, which could explain the preservation of the apoptotic function. The ratio of negative to positive charges in rDD could explain the lower stability of the EE mutant with respect to the WT in the MD simulations at 500 K.

### Radius of gyration

**Figure 3:**
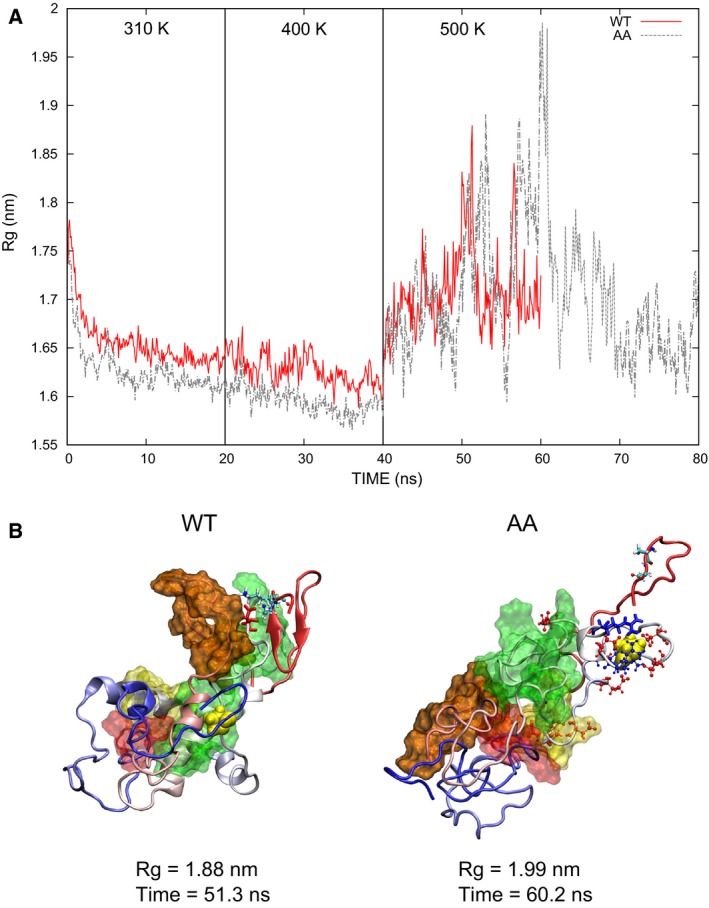
Radius of gyration. (A) The AA mutant reaches the largest Rg values at 500 K. (B) Structure with the largest elongation observed (AA mutant) contrasted with the largest elongation of the WT protein. In the WT, D98 (shown in Licorice, red) remains interacting with the C‐terminal lysines. On the other hand, charged residues in rDD seem to interact only with each other in the AA mutant (shown in CPK). R94 (shown in Licorice, blue) is close to the C terminus, but is even closer to negatively charged residues in rDD (shown in CPK, red).

While the structure of all proteins at all temperatures remains rather globular (Fig. 2), closer analyses show substantial differences in the compactness of the protein. The radius of gyration (Rg) shows that the WT protein remains compact, with values mostly below 1.8 nm in full agreement with previously reported results 9. Meanwhile, changes in the Rg are observed for the structure of the mutant variants of Bax at 500 K (Fig. S7). The largest value was found for the AA mutant, which reaches an Rg of over 1.95 nm. As this value was observed near the end of the simulation, 20 further nanoseconds of simulation were performed to look for larger changes (Fig. 3A). Indeed, an even larger value was reached at 200 ps in this extended simulation (Rg = 1.99 nm). This structure is depicted in Fig. 3B. In her Thesis, Boohaker reported increments in the Rg of a C‐terminal deletion mutant of Bax (DCT), even though the simulations performed were shorter (10 ns). However, further details of the resulting structures were not reported, other than the finding that DCT suffers a much larger conformational change than the WT protein or the mutants E69R, N73R, and E69R/N73R 15.

### Proximity of the C terminus to the core

**Figure 4:**
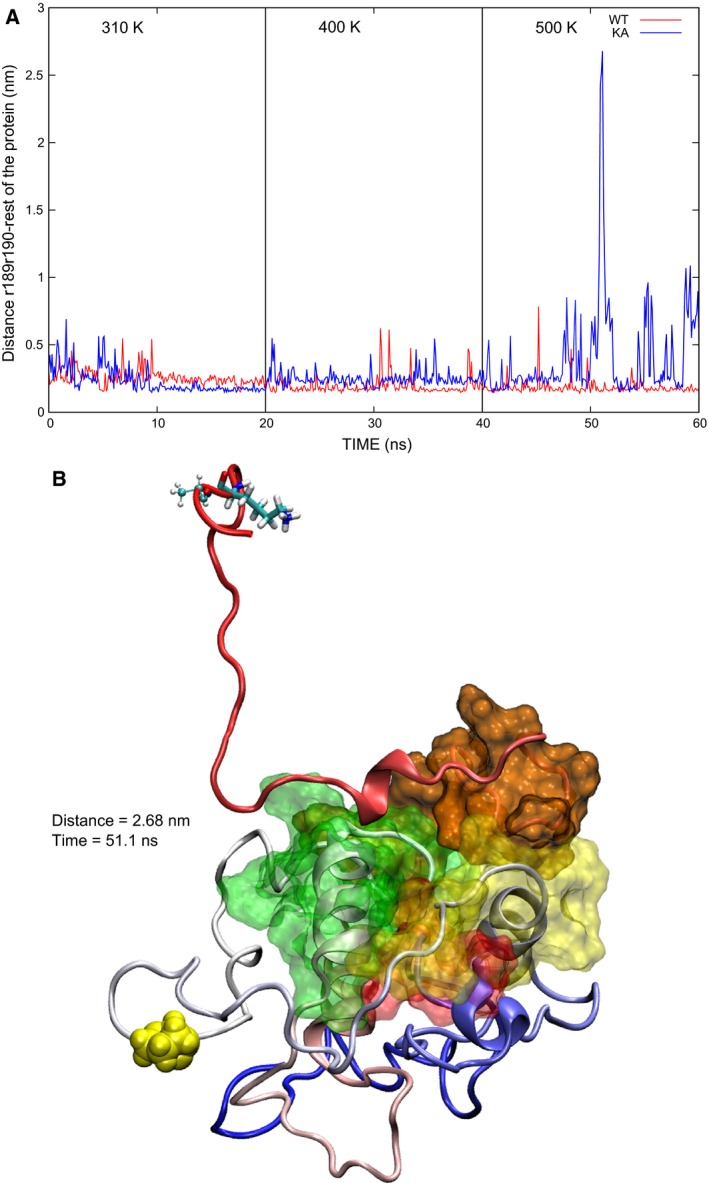
Distance between r189r190 and the core of the protein. (A) The maximum value (2.68 nm) is observed in the MD simulation of the KA mutant at 500 K. This value is more than two times larger than the maximum value observed in the WT. (B) The structure corresponding to the maximum value shows a large distance between r189r190 and P88 (shown in VDW). Besides, the core of the protein remains compact.

It has been suggested that the interactions of K189 and K190 provide a ‘clasp’ that prevents the separation of aH9 from the core, based on previously reported results 9. To estimate the effect of the mutations in this role, the minimum distance between residues 189–190 (r189r190) and the core of the protein (residues 1–169) was measured; larger values for the variants than for the WT would suggest that the C‐terminal lysines in the WT indeed help to hold aH9 in place. Interestingly, this distance remains always below 1 nm for the WT and reaches larger values for the mutant proteins (Fig. S8). The largest distance between r189r190 and the core of the protein is observed in the KA mutant at 500 K, with values above 2.5 nm (Fig. 4A). This behavior suggests that the mutations studied facilitate the separation of aH9 from the core of the protein. This conformational change seems to be prevented by K189 and K190 in the WT protein. To the author's knowledge, the conformation depicted in Fig. 4B is the first 3D model, where Bax reaches a conformation with such a large separation between aH9 and the hydrophobic groove, starting from the native conformation. Khaled *et al*. 18 produced a model, where aH9 was moved away from its original position by computationally rotating the psi angle of P168, which is located in Lp8‐9. They found that this modified conformation presented a more relaxed structure, which is consistent with the results mentioned above.

In the WT protein, most of the C‐terminal region remains close to the core of the protein, whereas a large part of the C‐terminal region in the AA mutant is separated at 500 K (Fig. S8). Also, the BH domains are less bundled together, as discussed below, and their helicity is lost in the AA mutant, as analyzed above. These unfolding events contribute to the structural changes observed for the AA mutant (Fig. 3B).

### Exposure of BH3

**Figure 5:**
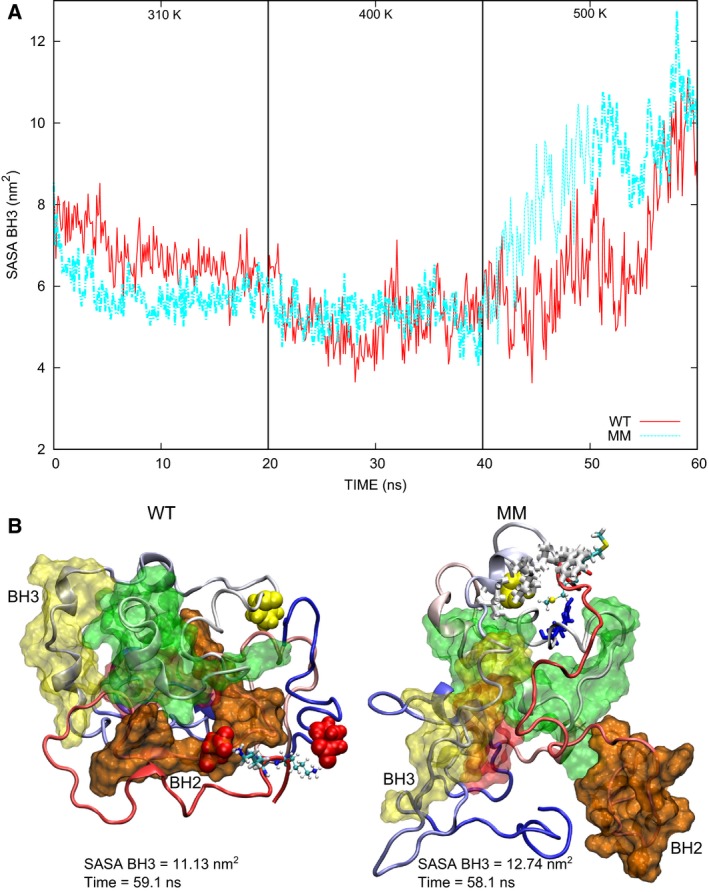
Solvent accessible surface area of the BH3 domain. (A) The values for the MM mutant are consistently above those for the WT at 500 K. (B) The final structures at 500 K reveal that the separation between BH2 and BH3 is larger in the MM mutant. Hydrophobic residues near the C terminus of the MM mutant and rDD (L76, M79, I80, A81, and W188) are depicted in white (Bonds representation). R94 is also nearby (shown in Licorice, blue), but does not seem to interact with the C terminus. E17 and D159 (shown in VDW, red) are found close to the C‐terminal lysines in the WT structure.

The solvent accessible surface area (SASA) decreases for all proteins at 310 K, mainly due to the approach of LpNt to the globule. SASA values also decrease at 400 K, where electrostatic interactions with rDD pull aH9 closer to the globule for the proteins with charged C‐terminal residues (WT, KA, AK and EE), while in the proteins with neutral residues (AA and MM) aH9 starts to move away from Lp3‐4 following the hydrophobic groove and reaches a conformation where the C terminus protrudes less than it does in the native one (Fig. 2). SASA values increase for all proteins at 500 K as the alpha helices unfold and the core unbundles. The largest SASA values at 500 K are found for the DPMs (MM, AA and EE) (Fig. S9). Interestingly, the SASA of BH3 increases in the mutant versions of Bax (Fig. S10). This is important because this domain is involved in the interaction between members of the Bcl‐2 family, particularly the autoactivation of Bax. This functionality requires the domain to be exposed. The largest exposure of BH3 is observed in the MM mutant at 500 K (Fig. 5A). Comparison of the structures with maximum BH3 SASA for the WT and MM mutant proteins reveals that BH2 shows a tendency to move away from the core and is less attached to BH1 in the mutant (Fig. 5B). Besides, BH2 is seen to lose contact with all other BH domains and is released from the Bcl‐2 core in other mutants as described below. In contrast, BH2 remains always close to the core in the WT (Fig. 2). The proximity of BH3 and BH2 seems to be stabilized by hydrophobic interactions involving residues in BH2, BH3 and BH4. These hydrophobic residues form a cluster in the MM mutant after 20 ns at 310 K. This cluster separates in the MD simulation at 500 K, where BH2 moves away from the N terminus of BH3 which is thus left exposed (Fig. S11). Arokium *et al*. 10 found that the MM mutant has a decreased binding capacity to mitochondria and little ability to release cytochrome c *in vitro*. These results are in agreement with the reduced stability of the MM mutant observed in the MD simulations.

### BH1‐4 unbundling

**Figure 6:**
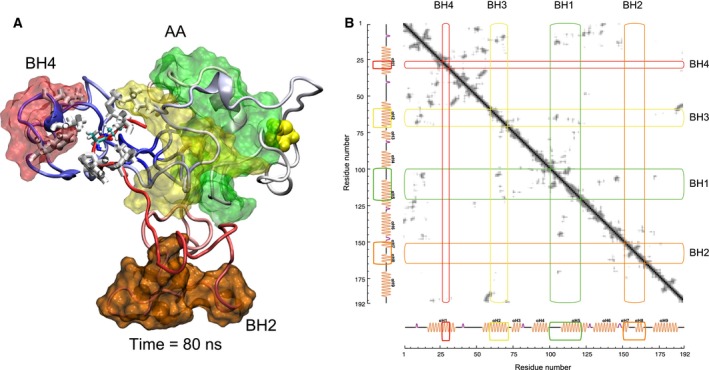
Unbundling of BH2 and BH4. (A) Final structure of the AA mutant after 40 ns at 500 K. Hydrophobic residues in BH4 and the C terminus close to L70 in BH3 (L25, L26, F30, L70, I187, W188, and M191) are depicted in white (Bonds representation). (B) Contact map for the structure in (A), BH2 and BH4 show a complete absence of contacts with other BH domains, whereas BH1 and BH3 are still in contact with each other.

The native conformation of Bax is globular and the BH domains show contacts (residues closer than 0.16 nm to each other) with two or even all three of the other BH domains, which results in a bundled conformation where all BH domains are not largely exposed (Fig. S12). On the other hand, the active conformation of Bax should expose BH3 and probably BH1 as well. The conformational changes that lead to the activation of Bax are therefore likely to modify the contacts between BH domains. Furthermore, the contact maps of the final structures from the simulations performed reveal remarkable differences between the WT and the mutant proteins. In the WT protein, all BH domains remain in contact with at least one other BH domain at all temperatures. The mutant proteins, in contrast, show unfolding of the bundled conformation. This unfolding is most noticeable in the AA mutant at the end of the extended simulation at 500 K, where BH2 and BH4 are completely separated from the original cluster of alpha‐helices and most of their surface is exposed (Fig. 6). The contacts between BH2 and either BH1 or BH3 decrease at high temperatures for most proteins. The same is observed for the contacts between BH4 and BH1. In contrast, BH3 remains close to either BH1 or BH4 in most cases (Fig. S12).

When aH9 is located in the hydrophobic groove, it holds BH1 close to BH2 as in the native fold. The mutations seem to increase the ability of aH9 to move, both away from the core and around the surface of the protein. Without the contribution of aH9 to keep close BH1 and BH2, the latter is able to escape from the core. This behavior is in agreement with the identification of BH2 as one of the least stable domains discussed above. Interestingly, BH4 is seen often in contact with the other BH domains, suggesting that BH4 has an important role in keeping the helical bundle structure of Bax. Additionally, hydrophobic residues in BH4 are found to interact with the C terminus of the AA mutant, thus helping to keep it close to the core (Fig. 6A). A detailed description of the role of BH4 in the structural stability of Bax is beyond the scope of this work and will be the subject of future research.

### Proximity of the C terminus to rDD

To further investigate the role of contacts between r189r190 and rDD, the distance between r189r190 and P88 (the central residue in rDD) was measured (Fig. S13). The values measured for the WT are always below 3 nm. The maximum value (4.6 nm) is observed for the KA mutant and coincides with the structure of maximum separation of r189r190 from the core (11.1 ns at 500 K, Fig. 4). The values for the AA mutant in the extended simulation at 500 K are also remarkably large (above 2.5 nm), which correlates with the Rg (Fig. 3) and the core unbundling observed in this simulation (Fig. 6). Of note, the values for the MM mutant are most of the time below 2 nm (Fig. 5B). M189/M190 are seen to interact with hydrophobic residues M79, I80, A81, A82, V83, V95, A96, A97, and F100 (Fig. S14). Nevertheless, several fluctuations larger than 1 nm appear for this protein at 500 K, which can be associated with the conformational changes that lead to the BH3 exposure observed in this simulation. Table S5 summarizes the charged residues in rDD within 0.7 nm of r189r190 in the final structures for all simulations. None are found at 500 K for KA and AA. In contrast, K189/K190 are found interacting with E17 and D159, which are not in rDD, but help to keep the C terminus close to the core in the WT. These results suggest that the mutations considered reduce or weaken the favorable interactions between r189r190 and rDD. This effect seems to correlate with a significant decrease in the structural stability of Bax.

## Conclusions

MD simulations at elevated temperatures may be used to study the unfolding pathway 22. The MD simulations reported here show interesting changes in the structural stability of Bax when mutations are performed in K189/K190 and the resulting structures are submitted to thermal stress. The loss of helical content is augmented in all mutants, but was most dramatic in the KA, AA, and EE mutants. Besides, the globularity of Bax was most affected in the AA mutant and the largest separation of the C terminus to the core was found in the KA mutant. The BH3 region is important for the interaction with other members of the Bcl‐2 family, hence its exposure should be part of the mechanism that leads to active Bax. All mutants show exposure of the BH3 domain; the largest occurs for the MM mutant. Moreover, the largest separations between BH domains are also seen in the mutant variants. The proximity of BH2 to the core seems to be largely maintained by the stability of aH9 inside the hydrophobic groove. In the EE mutant, the charges of r189r190 are negative, whereas they are positive in the WT. The stability of this variant is, however, not dramatically affected at 310 and 400 K. This behavior is explained by electrostatic interactions between E189/E190 and positively charged residues in rDD.

The conformational changes observed could be similar to the unfolding steps required to activate Bax. Furthermore, the conformations that show these changes might resemble active Bax. An existing model for the activation of Bax includes the displacement of aH9 as an early step 8. The MD simulations performed suggest that the release of BH2 would be the following step. Additionally, the separation of BH1 and BH3 would be among the last steps.

## Author contributions

JLRT conceived and designed the project, performed the simulations, analyzed the data and wrote the manuscript.

## Supporting information


**Appendix S1.** Figure 6 snapshot: AA_500K_EXT.pdb.

**Appendix S2.** Figure 2 snapshots zip file.

**Appendix S3**. Figures 2 and S11 snapshots zip file.

**Fig. S1.** Multiple sequence alignment of the C‐terminal end of Bax proteins from a variety of species.
**Fig. S2**. Alpha‐carbon root mean square deviation (RMSD).
**Fig. S3.** Alpha‐carbon root mean square fluctuation (RMSF).
**Fig. S4**. Time evolution of the secondary structure of the proteins at different temperatures.
**Fig. S5**. Time evolution of the helical content of the proteins at different temperatures.
**Fig. S6.** Time evolution of the mimimum distance between E189/E190 and R78/R94 (EE DPM).
**Fig. S7.** Time evolution of the radius of gyration (Rg) of the proteins at different temperatures.
**Fig. S8.** Distance between residues 189–190 and the protein without aH9 (residues 1–169).
**Fig. S9.** Time evolution of the solvent‐accessible surface area of the proteins at different temperatures.
**Fig. S10**. Time evolution of the solvent accessible surface area of the BH3 domain at different temperatures.
**Fig. S11**. The final structure from the MD simulations for the MM mutant at 310 K (A) and 500 K (B).
**Fig. S12**. Contact maps for the final structures of the proteins at different temperatures.
**Fig. S13.** Time evolution of the distance between C‐terminal residues 189 and 190 and residue P88, which represents the D68‐D98 region (rDD).
**Fig. S14.** Distance between M189/M190 and hydrophobic clusters 1 and 2.
**Table S1**. Details from the sequences of Bax proteins used for the alignment in Fig. S1.
**Table S2.** Box vectors of the original system for all simulations. The initial volume of each box was 473.151367 nm^3^.
**Table S3.** Composition of the systems used in the MD simulations.
**Table S4**. Mean percentage helix content.
**Table S5.** Charged residues in rDD within 0.7 nm of r189r190.
