# Supplementary material for: The importance of evolutionarily conserved C‐terminal basic residues for the stability of proapoptotic Bax protein
Source: FEBS Open Bio. 2016 Aug 25;6(10):976–86. doi: 10.1002/2211-5463.12096 (PMC5055034; doi:10.1002/2211-5463.12096)
Supplement: Supplementary file 4 — Fig. S1. Multiple sequence alignment of the C‐terminal end of Bax proteins from a variety of species. Fig. S2. Alpha‐carbon root mean square deviation (RMSD). Fig. S3. Alpha‐carbon root mean square fluctuation (RMSF). Fig. S4. Time evolution of the secondary structure of the proteins at different temperatures. Fig. S5. Time evolution of the helical content of the proteins at different temperatures. Fig. S6. Time evolution of the mimimum distance between E189/E190 and R78/R94 (EE DPM). Fig. S7. Time evolution of the radius of gyration (Rg) of the proteins at different temperatures. Fig. S8. Distance between residues 189–190 and the protein without aH9 (residues 1–169). Fig. S9. Time evolution of the solvent‐accessible surface area of the proteins at different temperatures. Fig. S10. Time evolution of the solvent accessible surface area of the BH3 domain at different temperatures. Fig. S11. The final structure from the MD simulations for the MM mutant at 310 K (A) and 500 K (B). Fig. S12. Contact maps for the final structures of the proteins at different temperatures. Fig. S13. Time evolution of the distance between C‐terminal residues 189 and 190 and residue P88, which represents the D68‐D98 region (rDD). Fig. S14. Distance between M189/M190 and hydrophobic clusters 1 and 2. Table S1. Details from the sequences of Bax proteins used for the alignment in Fig. S1. Table S2. Box vectors of the original system for all simulations. The initial volume of each box was 473.151367 nm3. Table S3. Composition of the systems used in the MD simulations. Table S4. Mean percentage helix content. Table S5. Charged residues in rDD within 0.7 nm of r189r190. [file FEB4-6-0976-s004.pdf]

Supplementary Data for:

The importance of evolutionarily conserved C-terminal basic residues for the stability of proapoptotic Bax protein

Jorge L. Rosas-Trigueros

Laboratorio Transdisciplinario de Investigación en Sistemas Evolutivos, SEPI de la ESCOM del Instituto Politécnico Nacional, Juan de Dios Bátiz y Miguel Othón de Mendizábal s/n, México DF, México

email: jlrosas@ipn.mx

```
Human               VTI-FVAGV--LTA-SL--TIW--KK-MG--- 192
Bovine              VTI-FVAGV--LTA-SL--TIW--KK-MG--- 192
Mouse               VTI-FVAGV--LTA-SL--TIW--KK-MG--- 192
Frog                VGV-FLAGV--LTA-SL--AIW--K--MS--- 221
Zebrafish           VGV-FLAGV--ITT-AL--VI---RK-M---- 192
Blue_catfish        VSI--IAAVA-FIA-AA--VYW--RR-TR--- 202
Hydra               YGV-FAAVI--LFAYKL---FF--RR------ 230
Tapeworm            IKP-LLSSLTFLIL-ALGTAFFLARA-RAFF- 622
G_psychrophila      RGQEYIDEL--LSM--L--RVN--RKHMETES 275
Termite             IAVITFCCFG-IVA--M--CVY-IRNNTH--- 352
                            .  :            :
```

Fig. S1. Alignment of the C-terminal end of Bax proteins from a variety of species. The conservation of positively charged residues (R, K) near the end is remarkable. Details from the sequences used are shown in Table S1. The Gonnet Matrix with NJ clustering, allowance for end gaps, 10 tree iterations and penalties 1, 0.05, and 1 for gap opening, extension and distances, respectively were used to produce the alignment in the Clustal W server [SR1].

[SR1] McWilliam, H., Li, W., Uludag, M., Squizzato, S., Park, Y. M., Buso, N., Cowley, A. P., Lopez, R. (2013). Analysis tool web services from the EMBL-EBI. *Nucleic acids research*, *41*(W1), W597-W600.

Table S1. Details from the sequences of Bax proteins used for the alignment.

Label	Species	Phylum/Class	UniProt Entry
**Human**	Homo sapiens	Phylum: Chordata<br>Class: Mammalia	Q07812
**Bovine**	Bos taurus	Phylum: Chordata<br>Class: Mammalia	O02703
**Mouse**	Mus musculus	Phylum: Chordata<br>Class: Mammalia	Q07813
**Frog**	Xenopus laevis	Phylum: Chordata<br>Class: Amphibia	Q98U13
**Zebrafish**	Danio rerio	Phylum: Chordata<br>Class: Actinopterygii	Q9I9N4
**Blue_catfish**	Ictalurus furcatus	Phylum: Chordata<br>Class: Actinopterygii	E3TCT7
**Hydra**	Hydra vulgaris	Phylum: Cnidaria<br>Class: Hydrozoa	T2MDZ0
**Tapeworm**	Echinococcus granulosus	Phylum: Platyhelminthes<br>Class: Cestoda	W6UL65
**G_psychrophila**	Glaciecola psychrophila	Phylum: Chordata<br>Class: Amphibia	K6ZK29
**Termite**	Zootermopsis nevadensis	Phylum: Arthropoda<br>Class: Insecta	A0A067QWK2

Table S2. Box vectors of the original system for all simulations. The initial volume of each box was 473.151367 nm^3.

Vector	X (nm)	Y (nm)	Z (nm)
**v1**	8.74658	0.00000	0.00000
**v2**	0.00000	8.74658	0.00000
**v3**	4.37329	4.37329	6.18477

Table S3. Composition of the systems used in the MD simulations.

Protein	Total atoms	Protein atoms	Water molecules	Na+ counterions
**WT**	46597	2977	14539	3
**AK**	46610	2965	14547	4
**KA**	46616	2965	14549	4
**AA**	46608	2953	14550	5
**EE**	46608	2963	14546	7
**MM**	46589	2967	14539	5

Table S4. Mean percentage helix content.

Protein	310 K	400 K	500 K
**WT**	58	44	27
**AK**	52	48	22
**KA**	57	51	20
**AA**	57	44	15 (60 ns), 9 (80 ns)
**EE**	54	43	21
**MM**	60	53	23

Table S5. Charged residues in rDD within 0.7 nm of r189r190. Charged residues outside rDD (shown in bold) are seen to interact with r189r190 in the final structures of WT and EE at 500 K. No charged residues in rDD are close to r189r190 in the final structure of AA in the extended simulation at 500 K.

Protein	0 ns	20 ns (310 K)	40 ns (400 K)	60 ns (500 K)
**WT**	R94	E75, R78	D84, D86, R89, E90	**E17, D159**
**AK**	R94	R78, D84	R78, D84	D68, D71, E75, R78
**KA**	R94	E75, R78	E75, R78, R89, R94	None
**AA**	R94	R94	E90, R94, D98	None
**EE**	R94	D84, E90, R94	R78, D84, D86, E90, R94	E69, D71, **R109, K119**
**MM**	R94	E75, R78, R94	D86, E90, R94	R94, D98

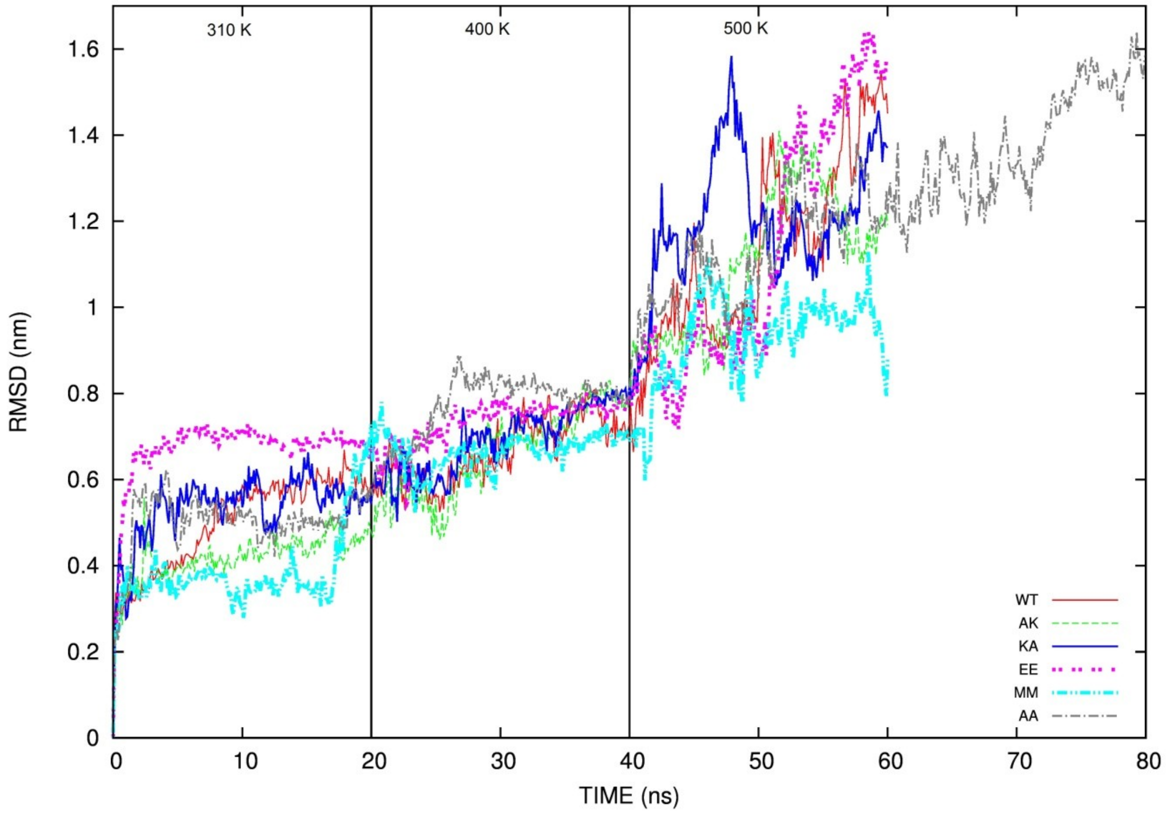

Fig. S2. Time evolution of alpha-carbons root mean square deviation (RMSD) at different temperatures.

A

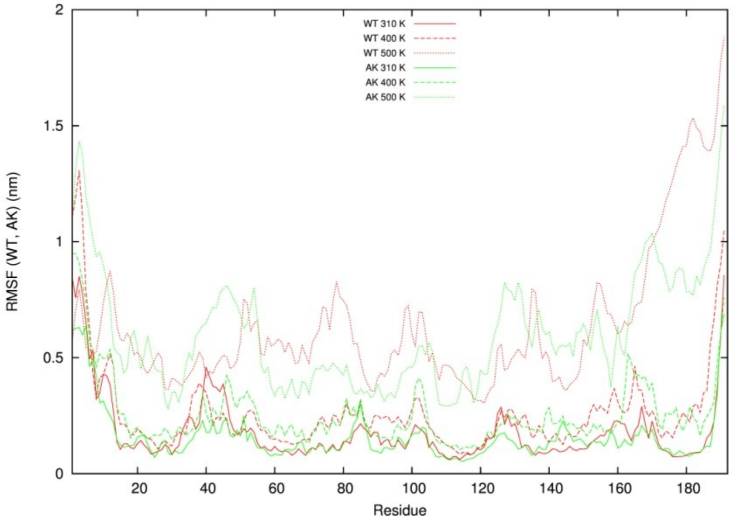

B

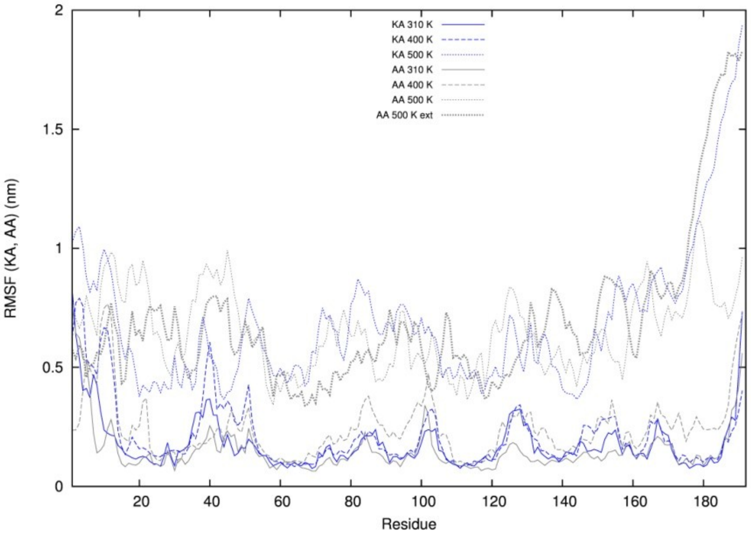

C

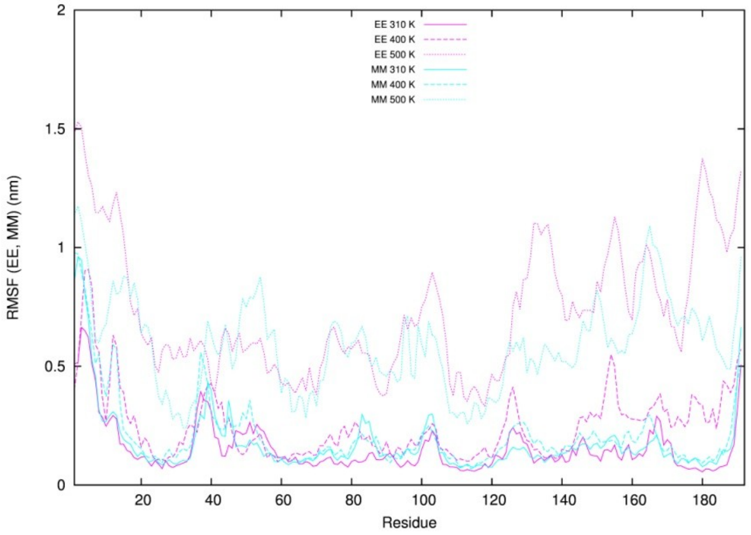

Fig. S3. The root mean square fluctuations (RMSF) of alpha-carbons coordinates. Values for WT and AK (A), KA and AA (B), and EE and MM (C) are shown as a function of residue number at different temperatures.

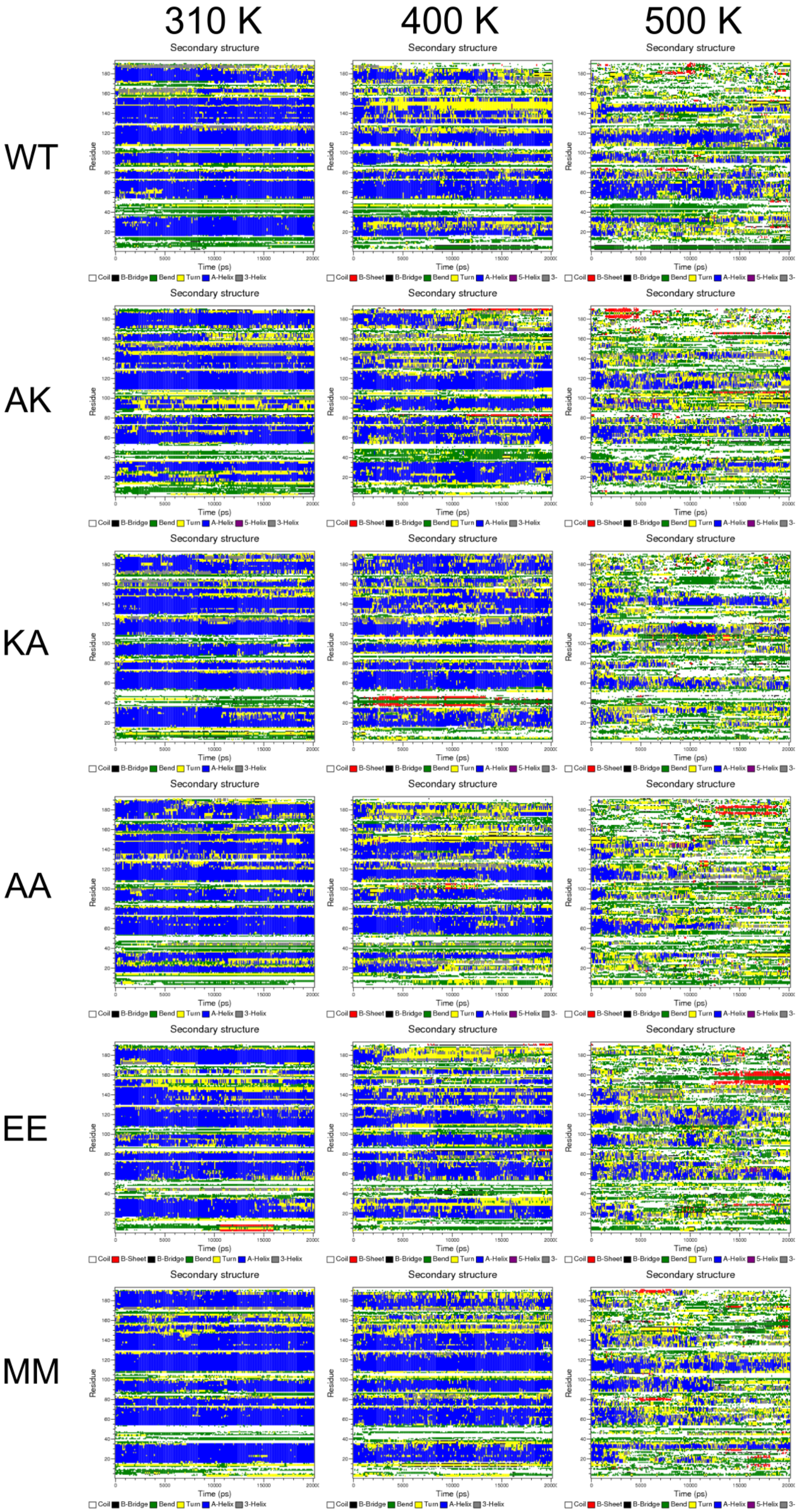

Fig. S4. Time evolution of the secondary structure of the proteins at different temperatures.

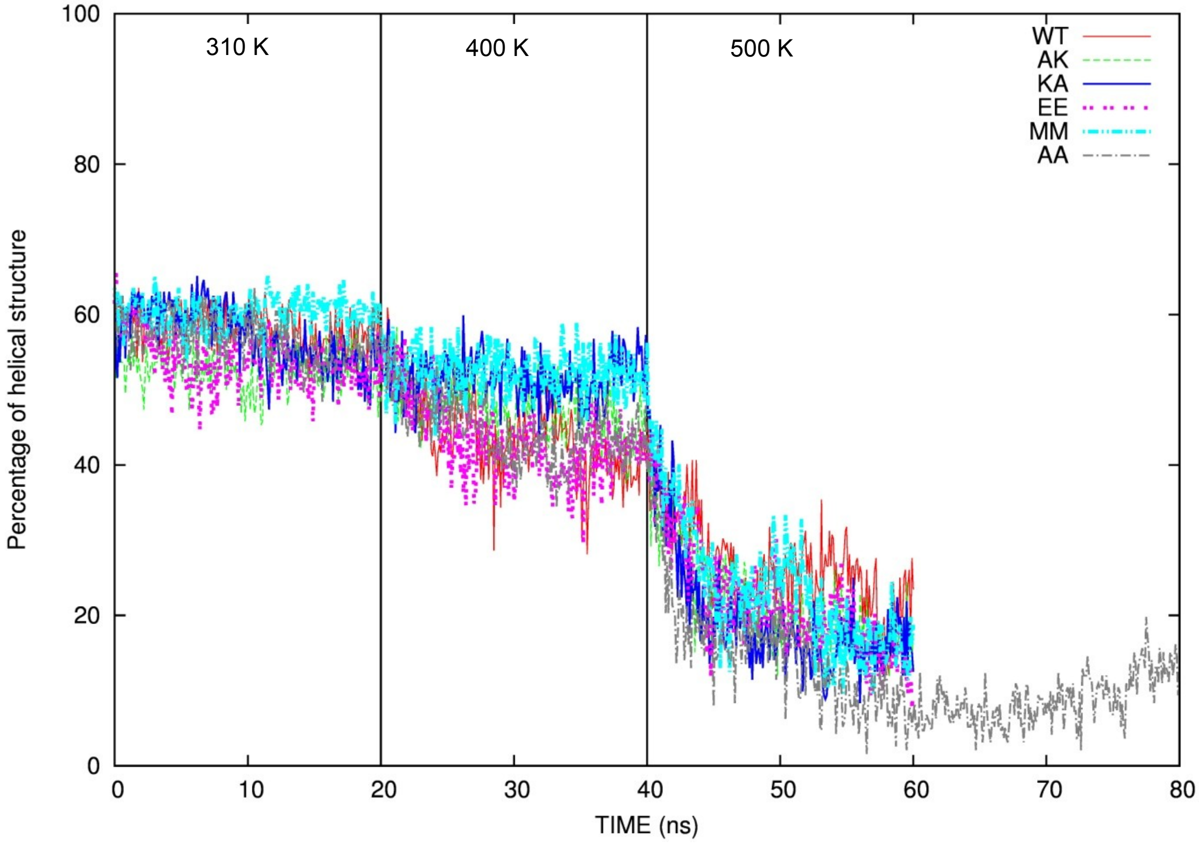

Fig. S5. Time evolution of the helical content of the proteins at different temperatures.

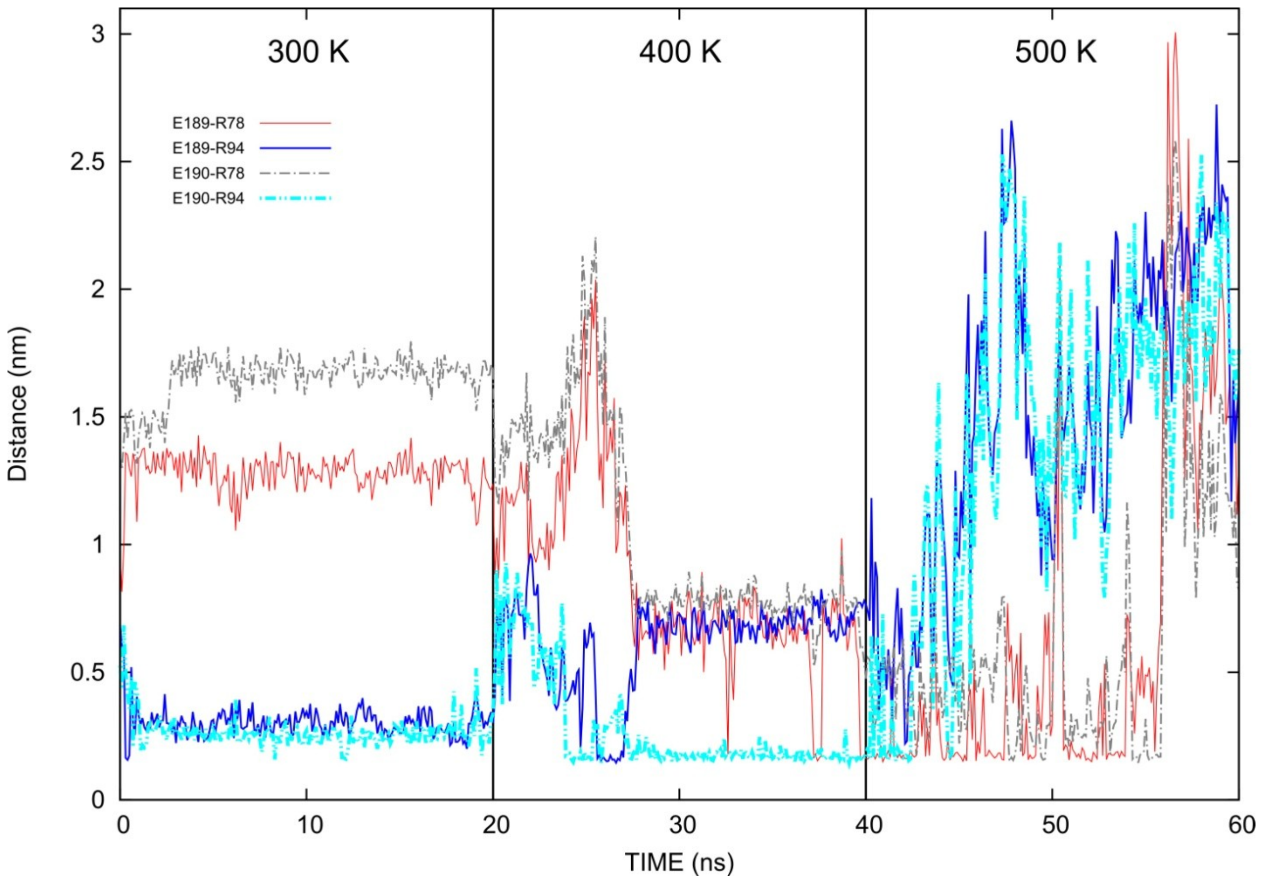

Fig. S6. Time evolution of the mimimum distance between E189/E190 and R78/R94 (EE DPM).

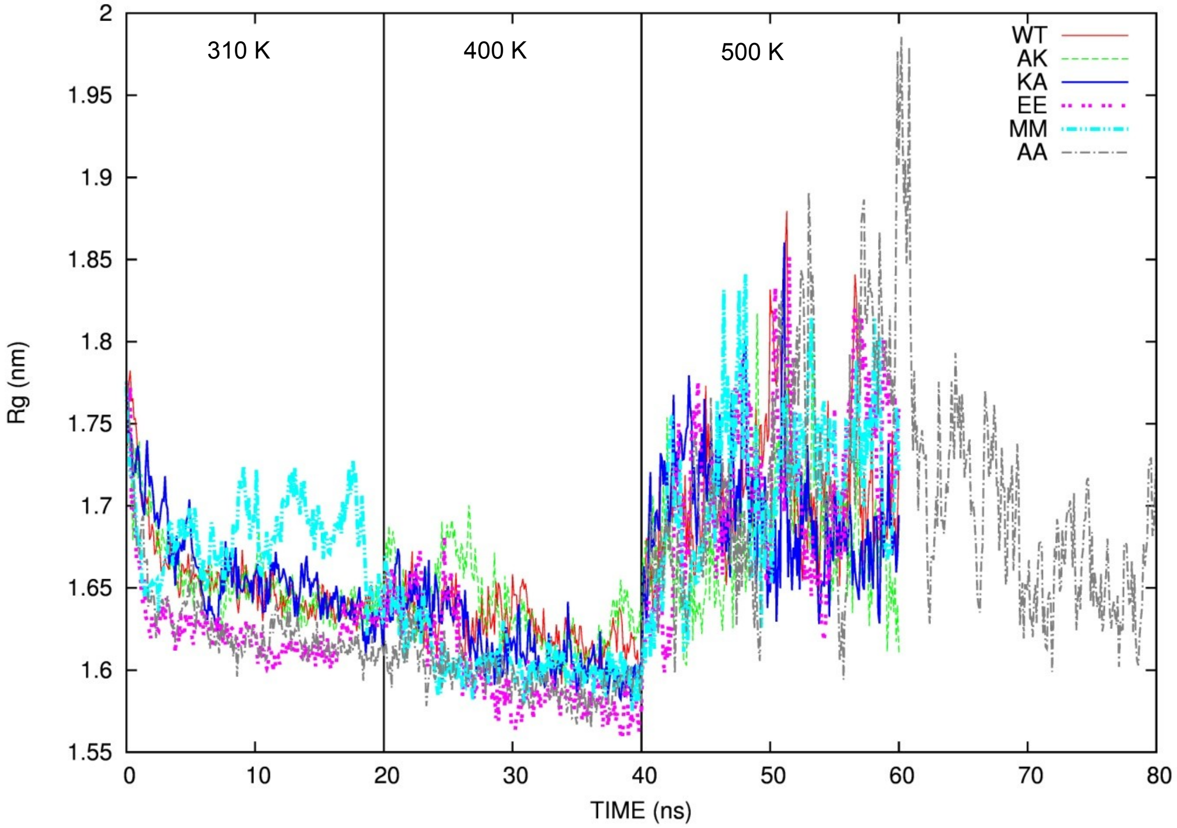

Fig. S7. Time evolution of the radius of gyration (Rg) of the proteins at different temperatures.

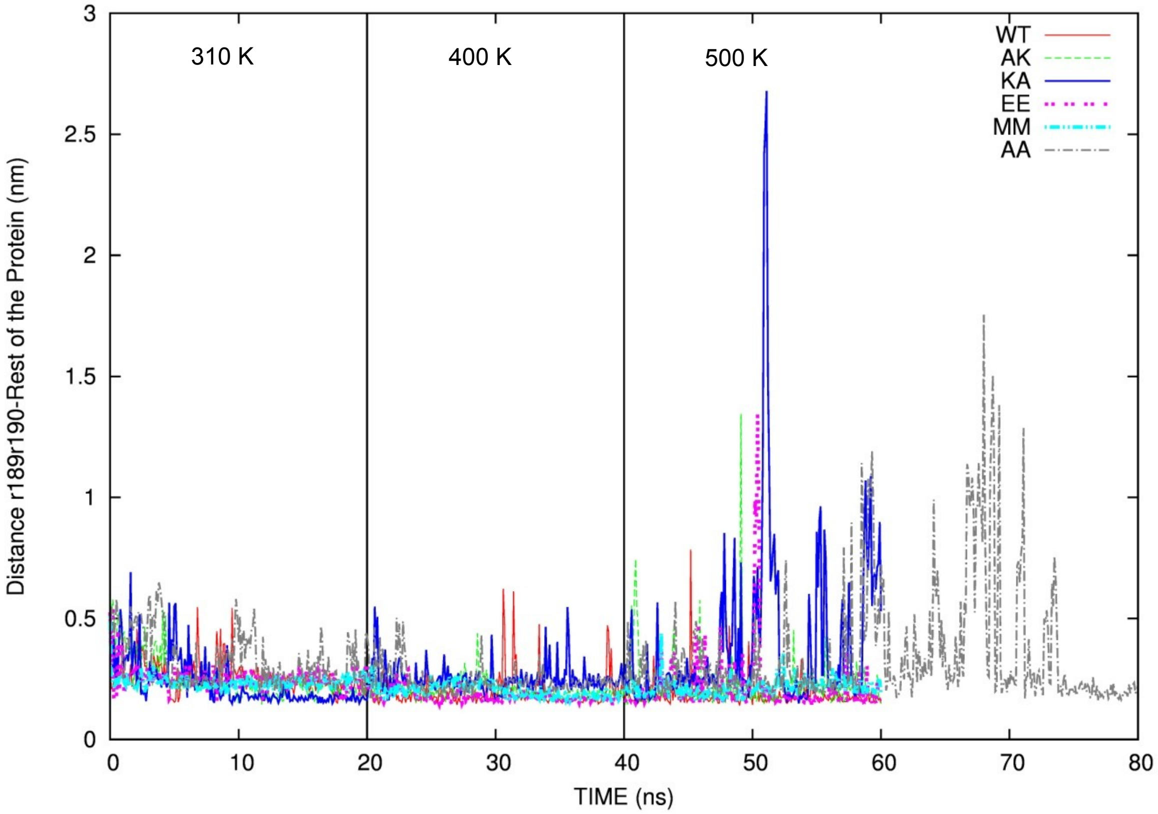

Fig. S8. Distance between residues 189-190 and the protein without aH9 (residues 1-169).

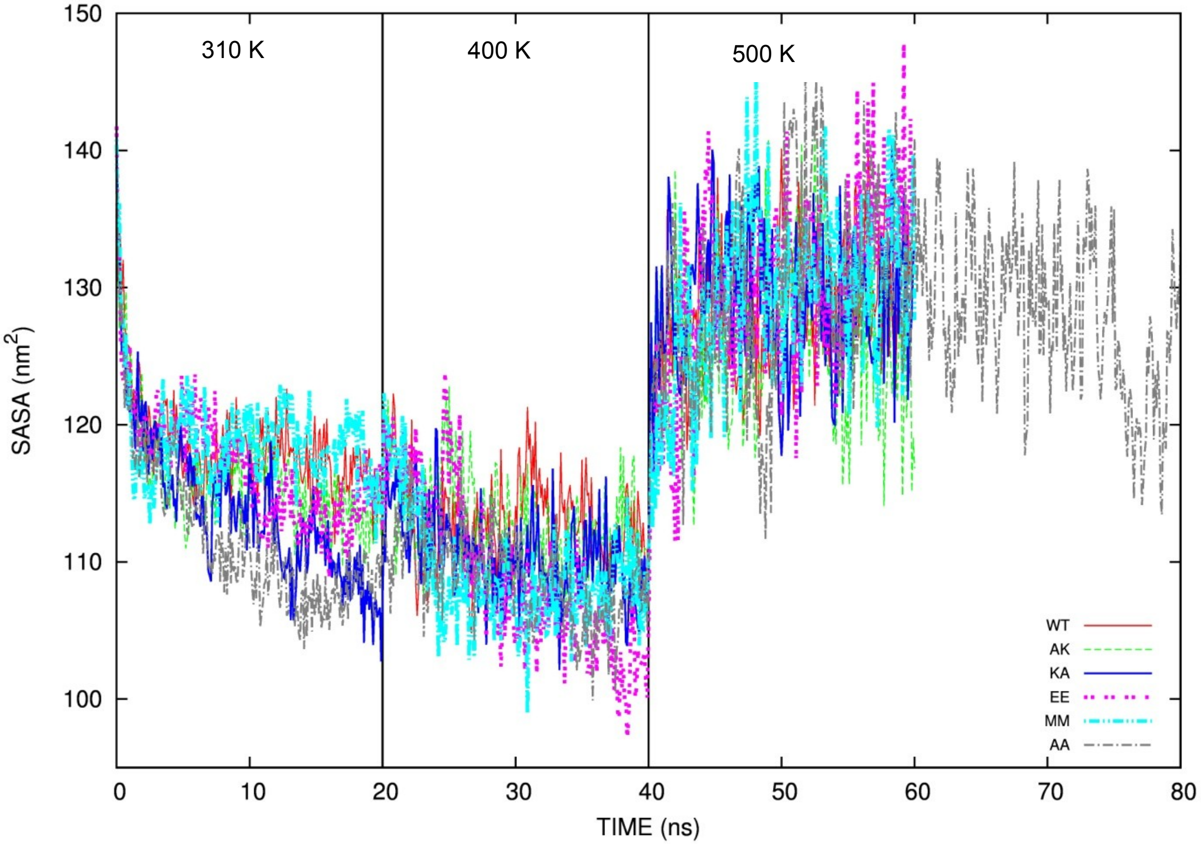

Fig. S9. Time evolution of the solvent accessible surface area of the proteins at different temperatures.

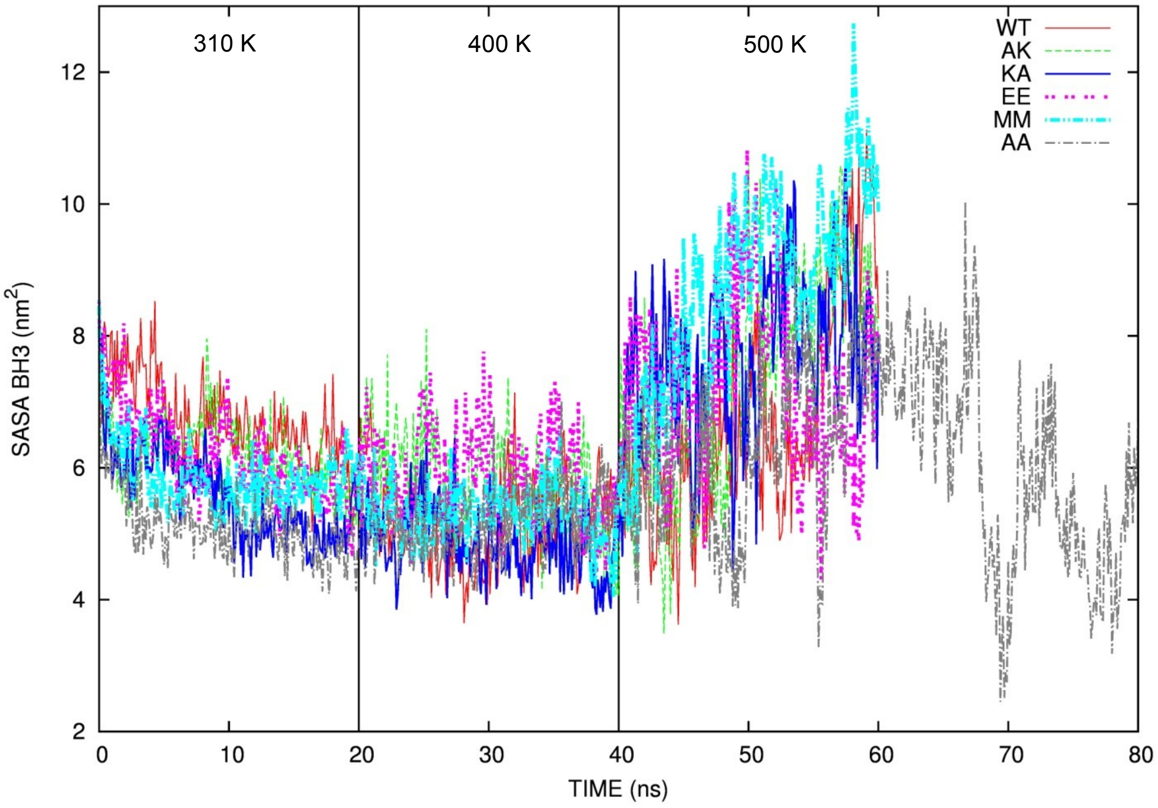

Fig. S10. Time evolution of the solvent accessible surface area of the BH3 domain at different temperatures.

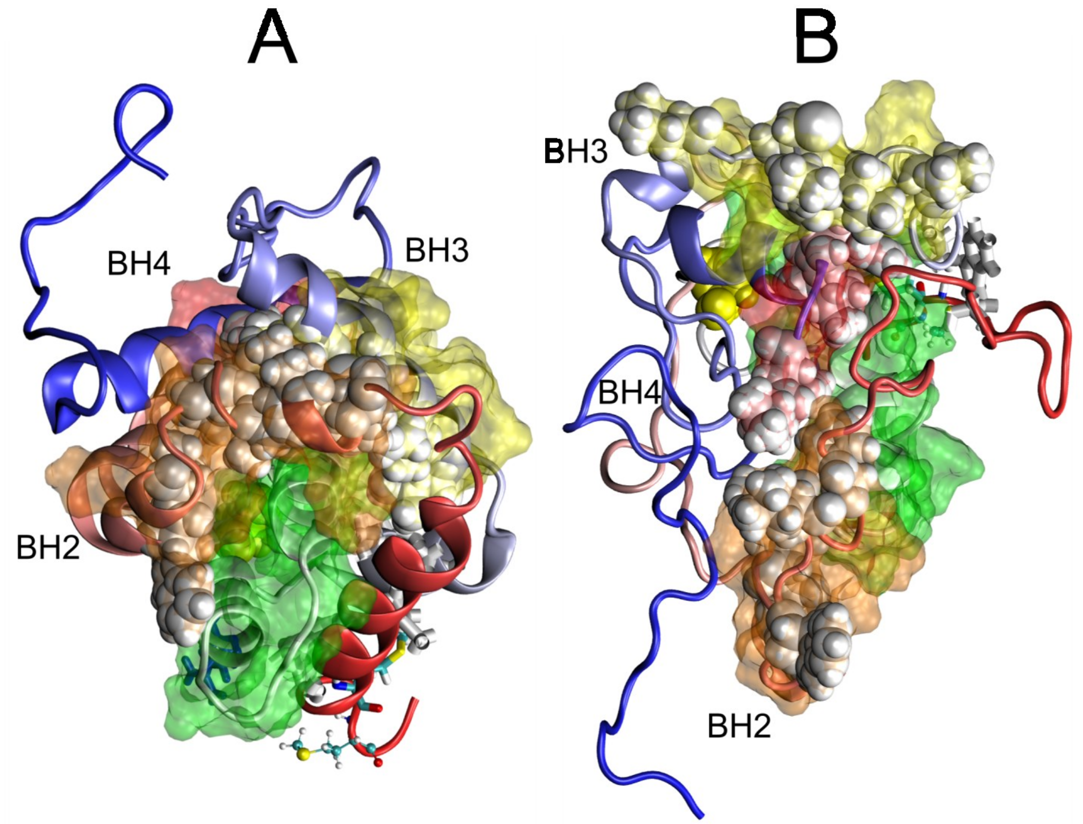

Fig. S11. The final structure from the MD simulations for the MM mutant at 310 K (A) and 500 K (B). The hydrophobic residues in BH2, BH3 and BH4 are sown in VDW (white). Color coding is the same as in Fig. 2 (main text).

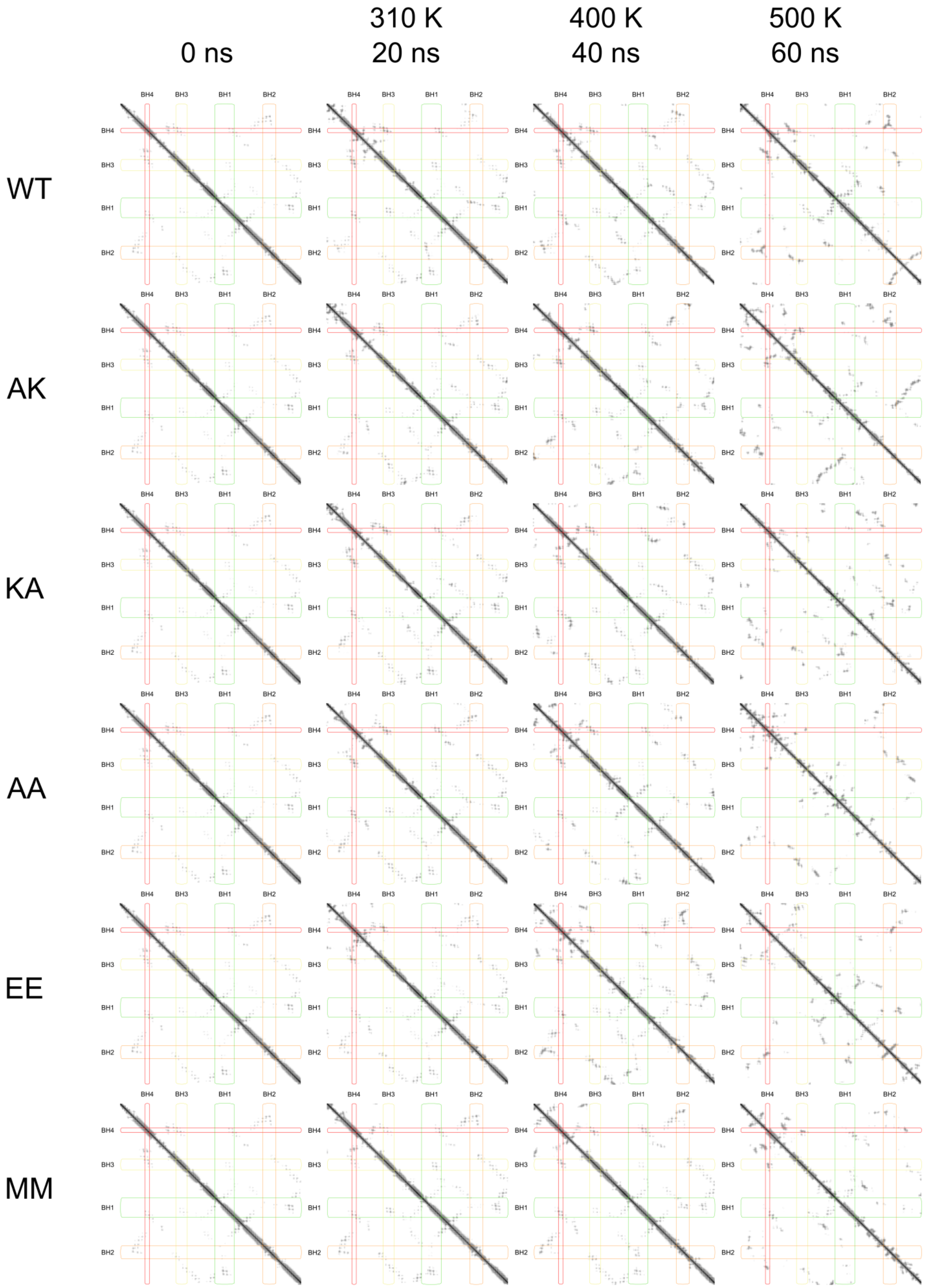

Fig. S12. Contact maps for the final structures of the proteins at different temperatures.

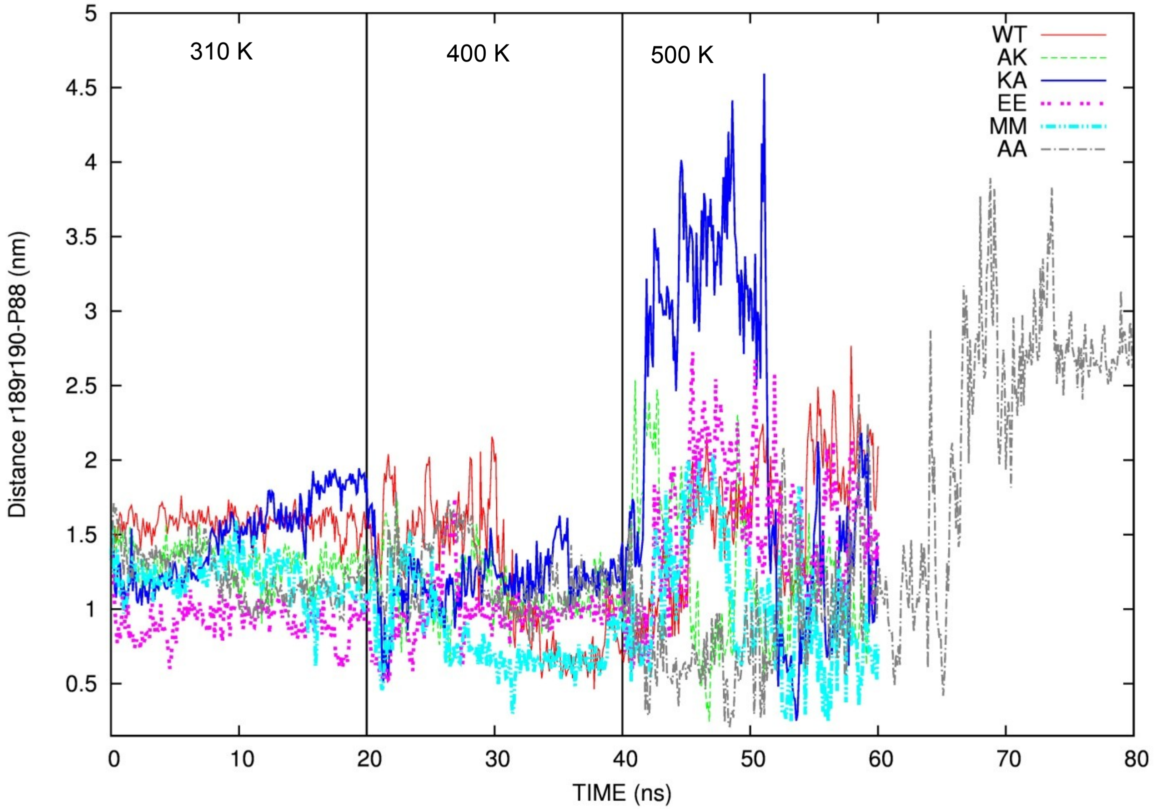

Fig. S13. Time evolution of the distance between C-terminal residues 189 and 190 and residue P88, which represents the D68-D98 region (rDD).

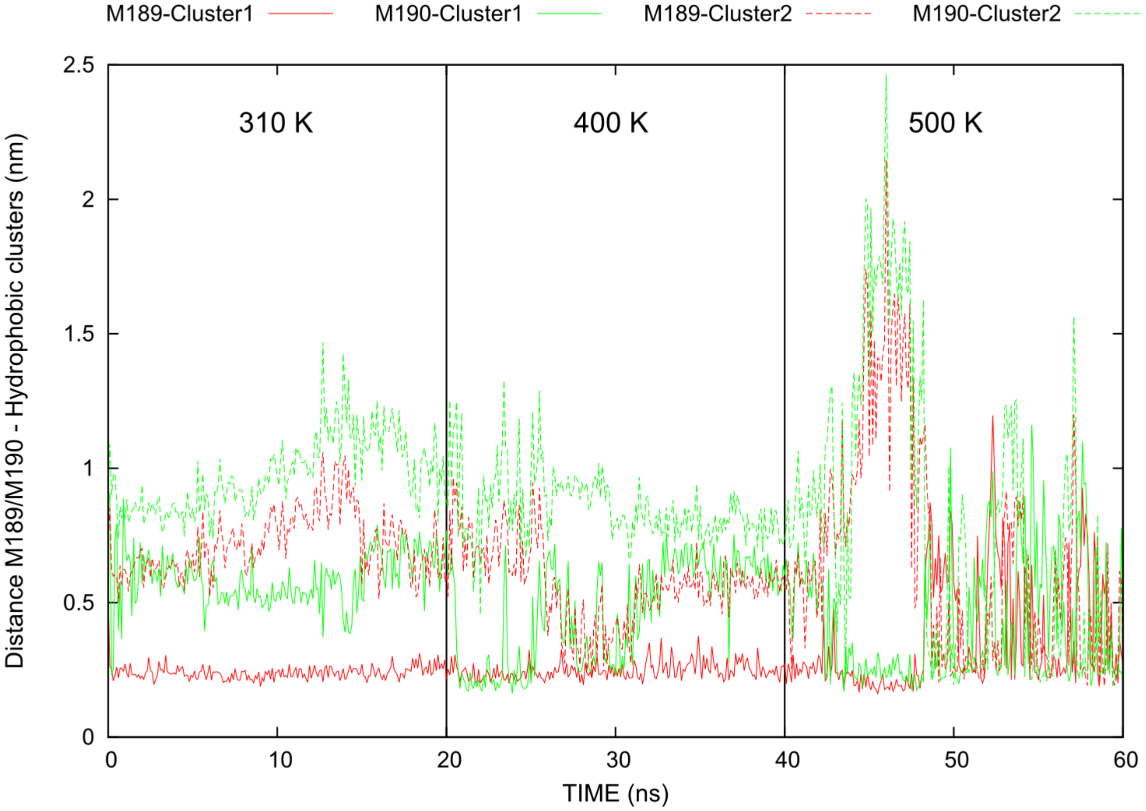

Fig. S14. Distance between M189/M190 and hydrophobic clusters 1 and 2. In the MM protein, M189 and M190 are close to hydrophobic clusters located in the proximity of P88. Cluster 1 includes M79, I80, A81, A82 and V83; cluster 2 includes V95, A96, A97 and F100.
